# Effect of Driving Pressure Modes on Microjet Dispersion Characteristics in Tissue-Mimicking Gels for Large-Volume Needle-Free Injection

**DOI:** 10.3390/gels12010095

**Published:** 2026-01-22

**Authors:** Dongping Zeng, Longsheng Luo, Linxing Luo, Wei Wang, Jiamin Li

**Affiliations:** 1 School of Energy and Power Engineering, Changsha University of Science and Technology, Changsha 410114, China; luols@csust.edu.cn (L.L.); ling@csust.edu.cn (L.L.); ondwei2001@csust.edu.cn (W.W.); 2 Hubei Key Laboratory of Waterjet Theory and New Technology, Wuhan University, Wuhan 430072, China; ljm2015@whu.edu.cn

**Keywords:** needle-free injection, agarose gel, driving mode, gel injection, dispersion characteristic

## Abstract

Needle-free injection (NFI) technology is a promising alternative to conventional syringe injection, as it mitigates needle-related complications and enhances patient compliance. However, achieving the controlled and efficient dispersion of larger-volume formulations (>1 mL) within tissues remains a significant challenge. This study presents a novel pneumatic NFI system that uses a two-phase driving mode to regulate driving pressure and duration with an ejection volume of 1.0–2.0 mL. The integrated pressure stabilization unit significantly reduces pressure fluctuations during the initial injection phase, generating a more stable and uniform spray distribution. It is designed to produce an ideal elliptical dispersion effect while eliminating splatter, enabling controlled large-volume delivery. Jet impact experiments were conducted to investigate the dynamic characteristics of microjets generated by conventional single-phase and novel two-phase driving modes. Furthermore, the influence of the driving mode on the dispersion behaviors of microjets in agarose gels was explored through high-speed imaging of gel injections. The results demonstrate that the two-phase driving mode produces a distinct two-phase jet pressure profile. Compared to the single-phase mode, the two-phase mode produced a significantly larger dispersion width at equivalent initial driving pressures. This promotes more uniform lateral drug distribution and achieves a higher percentage of liquid drug delivery in gels. Furthermore, favorable driving pressure combinations were identified for different volumes: (1.25–0.25) MPa for 1.0 mL, (1.25–0.50) MPa for 1.5 mL, and (1.50–0.50) MPa for 2.0 mL. This provides a practical basis for optimizing clinical parameters and advancing the development of controllable NFI systems.

## 1. Introduction

Needle-free injection (NFI) technology employs a high-speed microjet to penetrate the skin and deliver drugs to subcutaneous or intramuscular tissue. This approach has several advantages over conventional syringes, such as reducing the risk of needle-stick injuries and cross-contamination, and alleviating needle phobia. This could potentially improve patient compliance with frequent administration regimens, such as insulin therapy or vaccination [1,2]. The clinical efficacy and safety of NFI depend fundamentally on the dispersion pattern of the drug within the target tissue, which is directly governed by jet dynamics. Key parameters such as jet velocity, impact pressure and injection duration collectively determine the final dispersion depth and width, which has a significant impact on drug bioavailability, local tissue response and therapeutic outcome [3,4,5]. Considerable research has focused on understanding and optimizing NFI for small-volume delivery, typically below 0.3 mL. Foundational studies using experimental models and theoretical analyses have established relationships between jet parameters, tissue properties, and dispersion morphology [6,7]. However, there is a growing clinical demand for the reliable delivery of larger volumes exceeding 1.0 mL, driven by advances in biologics, vaccines, DNA-based therapies and local anesthetics [8,9]. The higher fluid momentum required for larger volumes poses a substantial engineering and biophysical challenge: if delivered under a constant driving force, it can result in excessive penetration depths, increased pain, and undesirable splash-back, whereby a portion of the formulation is not retained within the tissue [10,11]. Splash-back compromises dosing accuracy and poses a contamination risk.

Current commercial NFI devices predominantly operate on a single-phase, constant-pressure driving mechanism. While sufficient pressure ensures complete delivery at the cost of deep penetration and splash-back, lower pressure used to reduce trauma may lead to incomplete injection or shallow dispersion; this represents a critical trade-off in the approach [12,13]. This inherent limitation restricts the ability to adjust the dispersion geometry for different therapeutic needs. Although recent conceptual studies propose that modulating the energy of the jet during injection could optimize dispersion and reduce splash-back, systematic and quantitative investigations of programmable multi-phase driving modes for large-volume delivery remain limited [14,15]. The single-phase constant-pressure mechanism shows clear drawbacks when handling large volumes. Its jet dynamics, characterized by a simple peak-steady profile, cannot adapt to the dynamic changes in tissue resistance or differing penetration needs of the fluid front versus subsequent fluid during injection [16]. This results in inefficient energy distribution, whereby energy is either expended on excessive penetration or lost to splash-back due to inadequate lateral diffusion. These high-pressure points and elevated shear rates are closely associated with cellular damage and the mechanical activation of nociceptive nerve endings [17,18]. The associated shear stress and pressure fluctuations are potential key factors in tissue damage and elevated pain perception. Evaluations of existing commercial devices confirm that the incidence of splash-back and tissue injury increases significantly with larger-volume injections.

Confronted with the challenge of large-volume drug delivery, the core problem is balancing the energy required for initial penetration with that needed for subsequent dispersion. Simply increasing the driving pressure in a single-phase mode is often counterproductive, as high-velocity jets tend to form narrow, deep channels rather than a broad elliptical dispersion, and subsequent fluid is prone to high-speed retrograde ejection [19]. Research into alternative driving strategies has begun, including preliminary attempts at temporal jet energy modulation via mechanically spring-driven multi-stage systems or piezoelectric actuators [20,21,22]. A more direct and promising strategy is the modulation of the propellant driving pressure itself. A biphasic profile, comprising an initial high-pressure phase to breach the epidermal barrier and establish a channel, followed by a low-pressure phase to promote lateral dispersion at the target depth while minimizing terminal pressure buildup, is conceptually advantageous [23,24]. However, there is a significant gap exists between this concept and practical implementation. Current research lacks comprehensive quantitative data on how key operational parameters such as specific high- and low-pressure levels, switching criteria based on time or sensor feedback and phase duration ratios collectively determine final dispersion depth, width, retention efficiency and tissue stress. Moreover, numerous studies have been confined to injecting simple solutions into homogeneous materials, whilst research employing transparent, tissue-like gel models to visualize and quantify the complete dispersion kinetics of large-volume formulations under controllable pressure-driven delivery conditions remains insufficient.

This study introduces and evaluates a controllable two-phase driving mode for a pneumatic needle-free injection system, enabling controllable pressure-driven switching. The proposed driving mode aims to reduce penetration depth, increase lateral diffusion width, and minimize fluid backflow under large-volume injection conditions. First, the jet impact pressure and velocity characteristics are investigated under conventional single-phase and novel two-phase driving modes across varying driving pressures and ejection volumes through jet impact experiments. Then, high-speed imaging experiments of gel injections are conducted to investigate the penetration and dispersion patterns of jets in gels under both driving modes. Finally, by analyzing the dispersion characteristics formed by jets in agarose gels, the optimal two-phase pressure combinations and corresponding jet velocity features that maximize dispersion width while minimizing fluid loss and controlling penetration depth are further explored. The findings are expected to elucidate the dynamic characteristics and regulatory mechanisms of two-phase driven microjets in needle-free injection, thereby providing a foundation for advancing next-generation controllable, large-volume needle-free injection.

## 2. Results and Discussion

### 2.1. Dynamic Characteristics

**Figure 1:**
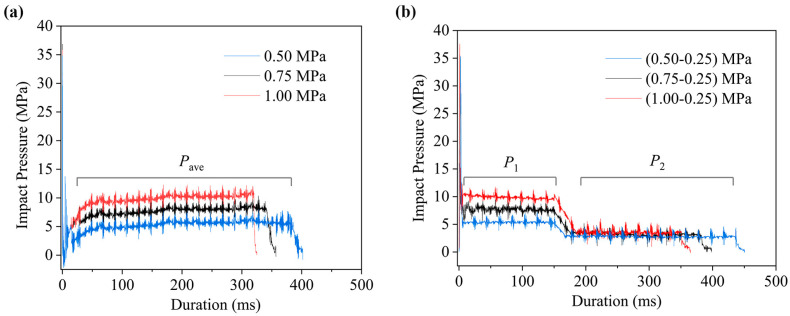
Impact pressure curves of the needle-free injection system under different driving modes and pressures. (**a**) Single-phase driving mode; (**b**) two-phase driving mode.

In the jet impact pressure test, a 0.17 mm nozzle and a 1.0 mL ejection volume were used to analyze the jet dynamic characteristics under different driving pressures. Figure 1 shows the impact pressure profiles of the system under both single-phase and two-phase driving modes. Figure 1a presents the results for single-phase driving pressures ranging from 0.50 MPa to 1.00 MPa. Upon exiting the nozzle, the jet rapidly reached a peak impact pressure of approximately 35 MPa, followed by a brief period of fluctuation before stabilizing. Comparison of the curves under different driving pressures reveals that the steady-state impact pressure increased with higher driving pressure, corresponding to values of 5.38 MPa, 7.71 MPa, and 9.92 MPa, respectively. Meanwhile, the total injection duration shortened accordingly, measuring 393 ms, 357 ms, and 325 ms. Notably, no clear correlation was observed between the initial peak pressure and the applied driving pressure.

Figure 1b illustrates the jet impact pressure curves under two-phase driving pressure conditions. The duration of the first phase for the 1.0 mL ejection volume was set to 150 ms in all impact experiments. Comparing the impact pressure curves of the two injection systems reveals that the controllable jet injection system under two-phase driving pressure exhibits a distinct two-phase jet impact pressure profile, with a clear inflection point during the transition from higher to lower pressure. Moreover, the inclusion of a pipeline pressure stabilization device resulted in reduced pressure fluctuation at the start of injection in the controllable system. Meanwhile, when the driving pressure of the first phase increases while the pressure of the second phase remains constant, the stable impact pressure of the first phase rises from 5.38 MPa to 9.92 MPa as the driving pressure increases from 0.50 MPa to 1.00 MPa, whereas the stable impact pressure of the second phase remains essentially unchanged. Moreover, the total jet duration under the two-phase mode exceeded that of a comparable single-phase injection.

**Figure 2:**
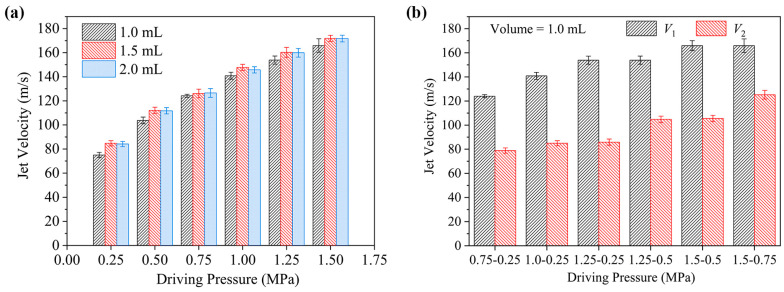
Jet velocity characteristics under different driving modes. (**a**) Single-phase mode with varying driving pressures and ejection volumes; (**b**) two-phase mode with varying driving pressures.

The jet velocity characteristics under various conditions are summarized in Figure 2, where *P*_1_ and *P*_2_ represent the average impact pressures for the first and second phases, respectively. Corresponding velocities *V*_1_ and *V*_2_ were calculated from these pressures using Equation (7). Figure 2a shows the velocity characteristics for the single-phase driving mode. For a fixed ejection volume of 1.0 mL, jet velocity exhibits a linear increase with driving pressure, rising from 84.14 m/s at 0.25 MPa to 171.75 m/s at 1.50 MPa. A driving pressure of 0.75 MPa produced a jet velocity of 124.22 m/s, meeting the minimum threshold required for effective needle-free injection [25]. When the ejection volume was increased from 1.0 mL to 2.0 mL under this same pressure (0.75 MPa), the jet velocity showed only a slight initial rise before stabilizing, with an overall variation in just 1.89%. These results demonstrate that for ejection volumes within the 1.0–2.0 mL range, any driving pressure exceeding 0.75 MPa is sufficient to satisfy the necessary jet velocity criteria.

Figure 2b presents the results for the two-phase driving mode. Increasing the first-phase driving pressure from 0.75 to 1.25 MPa produced a corresponding linear increase in the first-phase jet velocity (*V*_1_) from 124.22 m/s to 153.88 m/s. With the second-phase pressure constant at 0.25 MPa, the second-phase velocities (*V*_2_) were 78.88 m/s, 84.97 m/s, and 85.88 m/s, respectively. Notably, *V*_2_ shows a positive correlation with the first-phase driving pressure, attributable to the influence of residual high-pressure gas from the initial phase. Comparison between the two modes reveals that the high-pressure phase velocity in the two-phase mode is consistent with that of the equivalent single-phase condition. However, the low-pressure phase velocity is slightly higher than the velocity achieved by a single-phase injection at the same nominal low pressure. Furthermore, increasing the pressure levels for both phases results in a linear increase in their respective jet velocities. These findings demonstrate that pressure switching successfully generates the required high-low two-phase velocity profile in a controllable system, and that modulation of the driving pressures allows precise control over both the magnitude and duration of the jet velocities in each phase.

### 2.2. Gel Injection Process

#### 2.2.1. Single-Phase Driving Mode

**Figure 3:**
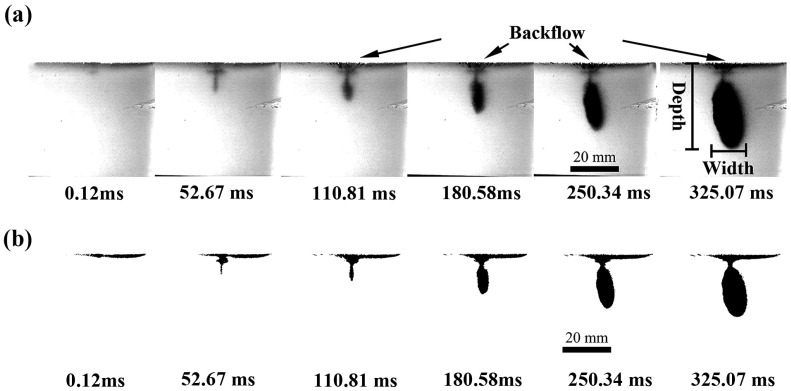
Injection process of a microjet within a gel under a single driving pressure of 1.0 MPa. (**a**) High-speed photographs of gel injection; (**b**) corresponding binarized images.

The jet penetration dynamics of a conventional pneumatic needle-free injection system into gelatin at a driving pressure of 1.0 MPa and an ejection volume of 1.0 mL are illustrated in Figure 3. As shown in Figure 3a, the process commences with the jet penetrating the gelatin surface until its kinetic energy is dissipated, forming an initial guiding channel. This is followed by a circular dispersion phase as the injected fluid spreads outward from the channel terminus. During the initial stage of injection (0.12 ms < t < 52.67 ms, where t denotes time after injection start), the penetration depth increases continuously while the width of the dispersion region remains largely constant. The remaining jet continued to infuse the gel, forming a semi-elliptical dispersion zone until injection ended (12.1 ms < t < 180.58 ms). During this phase, dispersion width kept increasing while penetration depth changed only slightly, as the jet expanded laterally near the channel end. In the final stage (180.58 ms < t < 325.07 ms), both penetration depth and dispersion width rose rapidly again. This suggests that under large-volume ejection conditions, the trailing part of the jet retained enough kinetic energy to extend the channel further inside the gel. It is also worth noting that pronounced splash-back of the jet onto the gel surface was observed after t > 110.81 ms. This phenomenon is attributed to the fact that during the later injection phase, the volume flow rate of the permeating gel persistently exceeds the rate at which stable pore channels form within the gelatin medium. This occurs due to differences in the mechanical properties of the gel, specifically its viscoelasticity and isotropy, compared to those of real skin.

**Figure 4:**
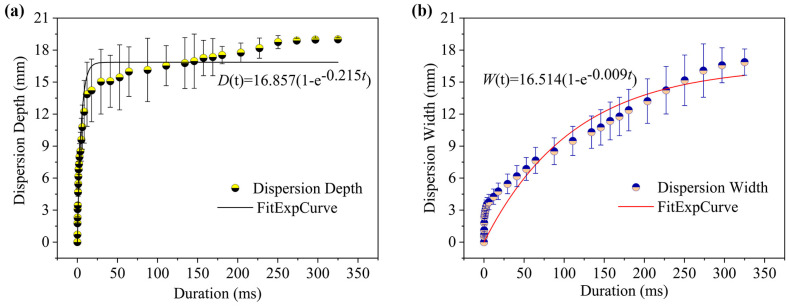
Variation in dispersion characteristics with injection duration at a single-phase driving pressure of 1.0 MPa. (**a**) Dispersion depth; (**b**) dispersion width.

To quantitatively analyze the dispersion characteristics under different injection conditions, the temporal evolution of dispersion depth and width were investigated. As shown in Figure 3b, the jet shape within the gels was binarized using MATLAB software (R2022a), enabling quantitative extraction of depth and width metrics from the processed image sequences. Figure 4 illustrates the temporal evolution of dispersion depth and width under these injection conditions. As depicted in Figure 4a, the dispersion depth within the gel rapidly increased to 15.04 mm within the first 30 ms post-injection, continuing to rise thereafter. It reached a maximum value of 18.75 mm at approximately 250.34 ms before stabilizing. As depicted in Figure 4b, the dispersion width exhibits distinct characteristics: it similarly increases rapidly to 5.46 mm within the initial 30 ms, after which the rate of increase slows but maintains steady growth until the end of injection, ultimately reaching a maximum width of 16.88 mm. According to existing needle-free injection kinetics studies, the dispersion width typically remains largely unchanged during the initial phase of rapid penetration depth [26]. The observed discrepancy may relate to differences in mechanical properties between the gel model material and actual skin tissue, thereby altering the lateral diffusion behavior of the jet.

Based on this, this study used the saturation exponential function Equation (1) proposed by Simmons and Taberner et al. [27,28] to fit experimental data:

*D*(*t*) = *D*_max_ (1 − *e*^−*kt*^) (1)

where *D*_max_ represents the final dispersion depth or maximum dispersion width, and *k* is the rate constant. The model is frequently employed in infusion studies, as it captures the process of approaching the maximum dispersion size (*D*_max_) asymptotically. The rate constant k reflects the combined effect of the injection driving force and tissue/gel resistance. In this context, a higher k value indicates faster filling of the dispersion cavity.

The fitted expression for dispersion depth as a function of injection time is:

*D*(*t*) = 16.857 (1 − *e*^−0.215t^) (2)

Equation (2) shows a high level of agreement with the experimental data. With a Chi-Sqr convergence tolerance of 1 × 10^−9^, the adjusted *R*^2^ value reached 0.941. The fitting results suggest that Equation (2) accurately describes the temporal variation in dispersion depth. The fitted value for parameter *D*_max_ was 16.857, which closely matched the average dispersion depth (17.288 mm) observed experimentally after 30 ms. This corroborates the conclusion of Simmons and Taberner that the maximum injection depth can be reliably predicted by this model, which reflects depth evolution during the injection process effectively.

The same fitting method was applied to the dispersion width data:

*W*(*t*) = 16.514 (1 − *e*^−0.009t^) (3)

The adjusted *R*^2^ value for the dispersion width fit is 0.887. The primary deviation in the fitting results occurs within the first 30 milliseconds after initiation of injection, during which phase the model fails to fully capture the initial rapid expansion process. Consequently, there is a discrepancy between the measured maximum dispersion width and the fitted parameter *W*_max_. However, beyond 30 ms, the fitting performance improves significantly, indicating that Equation (3) effectively predicts the dispersion width trend following the initial transient phase.

#### 2.2.2. Two-Phase Driving Mode

**Figure 5:**
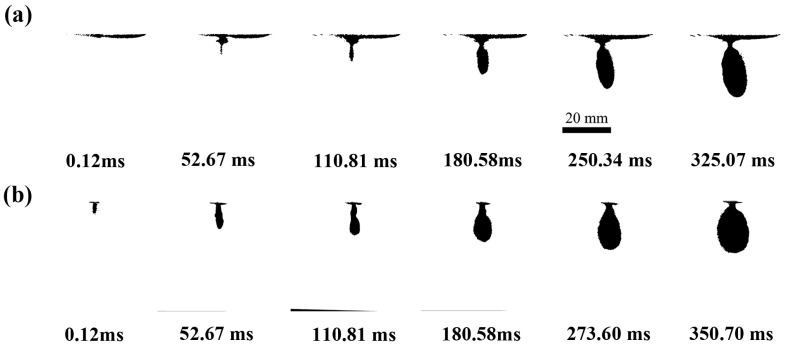
Binarized images of microjet injection into gel: (**a**) under a single driving pressure of 1.0 MPa, (**b**) under a two-phase driving pressure of (1.00–0.25) MPa.

Figure 5b illustrates the entire jet injection process into the gel under the two-phase drive mode (1.00–0.25 MPa) with an ejected volume of 1.0 mL, to elucidate the differences in jet penetration and diffusion processes within the gel under the two drive modes (Figure 5). This process can be divided into three sequential stages. During the initial phase (0 ms < t < 52.67 ms), the dispersion depth continuously increased while the dispersion width remained largely unchanged. Entering the second phase (52.67 ms < t < 180.58 ms), both the depth and the width increased, with the width increasing significantly more than the depth. This resulted in the formation of a semi-elliptical dispersion region within the gel. During the final stage (180.58 ms < t < 350.70 ms), the dispersion width expands rapidly once more while the penetration depth stabilizes. This stabilization of the depth is due to the pressure switching to a lower second phase (0.25 MPa) at 150 ms. At this point, although the jet energy is insufficient to continue penetrating the tissue, it remains effective in promoting lateral diffusion. Upon entering the gel, the fluid rapidly penetrates forward along the formed guiding channel, with the depth increasing rapidly. Subsequently, the jet expands outward from the channel’s terminus, gradually increasing in dispersion width until, upon completion of injection, a near-elliptical dispersion zone is formed. Compared to the single-phase drive mode (Figure 5a), no jet splash back was observed on the gel surface during the two-phase injection process. Furthermore, the dispersed region that formed within the gel was wider and closer to the desired ideal elliptical shape for needle-free injection. Elliptical dispersion profiles are often considered advantageous for drug delivery because they increase the absorption surface area within target tissue layers [18]. These phenomena suggest that the two-phase mode improves the efficiency of fluid delivery and increases the dispersion area, while also enabling effective control of the dispersion morphology. From a drug delivery perspective, this mode exhibits superior performance by potentially facilitating enhanced tissue absorption.

**Figure 6:**
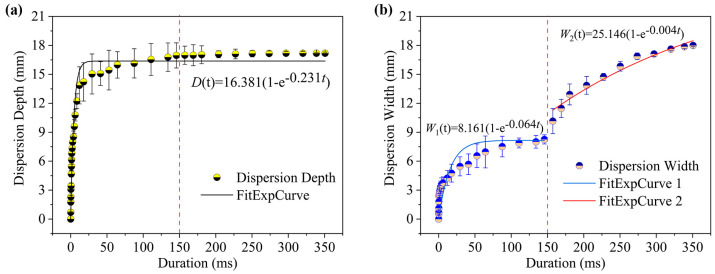
Variation in dispersion characteristics with injection duration under a two-phase driving pressure of (1.00–0.25) MPa. (**a**) Dispersion depth; (**b**) dispersion width.

Figure 6 presents the quantitative results for the dispersion characteristics under the two-phase driving mode. Figure 6a shows that the dispersion depth 150 ms after injection is similar to that observed in the single-phase mode. Following pressure switching, the depth stabilizes at approximately 16.73 mm, indicating entry into a stable drug delivery phase. During this phase, the fluid deposits at a specific depth and undergoes extensive lateral diffusion. Figure 6b shows how the dispersion width changes over time. This likewise has three distinct phases. The first phase (approximately 6 ms) sees the width expand rapidly to 3.76 mm. This corresponds to the formation of the initial channel. The second phase (6 ms < t < 150 ms) sees the width gradually increase from 3.76 mm to 8.06 mm. This occurs during the primary fluid transport period. During this time, both the depth and the width increase, but at different rates. The third phase (t > 150 ms) sees the width increase almost linearly from 10.16 mm to 18.15 mm. This is primarily driven by the low-pressure phase, which causes the jet to expand laterally.

The saturation exponential model was similarly applied to fit the dispersion depth data under two-phase driving:

*D*(*t*) = 16.381 (1 − *e*^−0.231t^) (4)

Equation (4) showed an excellent fit, with an adjusted *R*^2^ value of 0.857. The parameter *D*_max_ (16.381 mm) closely matched the average depth at 30 ms (16.729 mm). Compared to the single-phase fitting results, this model showed good agreement with the trend of depth variation under two-phase driving, thus confirming its predictive reliability in this context.

Due to the distinct two-phase variation in dispersion width observed, a piecewise fitting approach was adopted. The expressions for the first (t ≤ 150 ms) and second (t > 150 ms) phases are given by Equations (5) and (6), respectively.

*W*_1_(*t*) = 8.161 (1 − *e*^−0.064t^) (5)

*W*_2_(*t*) = 25.146 (1 − *e^−^*^0.004*t*^) (6)

The adjusted *R*^2^ values for the first and second phases of fitting were 0.852 and 0.958, respectively. The notably higher quality of fit demonstrated in the second phase indicates that segmented modeling accurately captures dynamic variations in dispersion width under dual-phase driving. Consequently, it provides a reliable foundation for trend forecasting, reflecting the increased complexity and control requirements of novel driving modes [9,11].

A comparative study of the penetration and diffusion evolution patterns of microjets within gels under single-phase and two-phase drive conditions reveals distinct differences between the two modes. The single-phase drive involves a three-stage process of penetration, diffusion and splash back. Penetration depth is well described by a saturation exponential function. However, this mode is susceptible to jet splash back, resulting in a final dispersion morphology that deviates from the ideal elliptical shape [24]. In contrast, the two-phase driving mode enables precise control over jet dispersion via active high-low pressure switching. It effectively eliminates splash back, promotes stable fluid deposition at the target depth, enhances lateral diffusion and produces a dispersion profile closer to an ellipse. This significantly improves fluid delivery and distribution efficiency. Furthermore, while the saturation index model accurately predicts penetration depth for both modes, modeling dispersion width in the two-phase mode requires segmented fitting functions to achieve higher precision. These findings demonstrate the unique advantages of the two-phase driven mode in preventing drug loss, controlling dispersion morphology and promoting tissue absorption. This highlights its significant potential for advanced, needle-free drug delivery applications.

### 2.3. Dispersion Depth in Gel Injection

**Figure 7:**
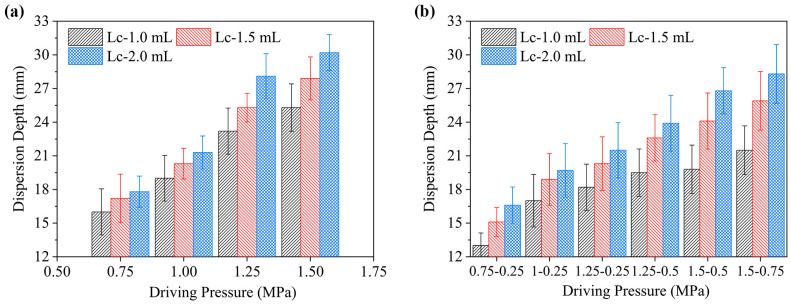
Dispersion depth characteristics of two driving modes in a needle-free injection system under varying driving pressures and ejection volumes. (**a**) Single-phase driving mode; (**b**) two-phase driving mode.

Figure 7 shows how driving pressure affects the final dispersion depth within the gel under single-phase and two-phase drive modes across an ejection volume range of 1.0 to 2.0 mL. Figure 7a shows that, when the injection volume is 1.0 mL, increasing the single-phase driving pressure from 0.75 MPa to 1.50 MPa results in an increase in dispersion depth from 16.1 mm to 25.3 mm. This demonstrates a clear positive correlation. A similar trend is observed for 1.5 mL and 2.0 mL volumes. Furthermore, at a constant driving pressure of 1.00 MPa, the dispersion depth increases from 18.9 mm to 21.3 mm as the ejection volume increases from 1.0 mL to 2.0 mL. Therefore, under the single-phase driving mode, the dispersion depth exhibits positive correlations with both driving pressure and ejection volume. As depicted in Figure 7b, for a 1.0 mL ejection volume under the two-phase mode, dispersion depths of 13.02 mm, 17.06 mm, and 18.21 mm correspond to driving pressure combinations of (0.75–0.25) MPa, (1.00–0.25) MPa, and (1.25–0.25) MPa, respectively. Notably, these depths are all smaller than those achieved under corresponding single-phase pressures (0.75, 1.00, and 1.25 MPa). This indicates that for the same initial (first-phase) pressure, the two-phase mode yields a reduced dispersion depth compared to the single-phase mode.

Figure 7b shows the dispersion depth of the 1.0 mL jet within the gel. Increasing the first-phase pressure from 1.00 MPa to 1.25 MPa correspondingly enhanced the depth. However, when the first-phase pressure was held at 1.25 MPa and the second-phase pressure was increased to 0.50 MPa, the dispersion depth remained largely unchanged. This suggests that, for 1.0 mL volumes, the two-phase mode effectively controls the dispersion depth. For ejection volumes of 1.5 mL and 2.0 mL, however, this control diminishes markedly, accompanied by pronounced jet splash back phenomena. These suggest that when the ejected volume exceeds 1.0 mL the two-phase mode requires higher driving pressures. To address this issue, four additional pressure combinations within the 1.0–2.0 mL range were introduced to determine the favorable configuration: (1.25–0.25) MPa, (1.25–0.50) MPa, (1.50–0.50) MPa, and (1.50–0.75) MPa.

Figure 7b shows that as the second-phase pressure increases from 0.50 MPa to 0.75 MPa, the dispersion depth rises significantly and jet splash back occurs again. This is because, although the 0.75 MPa pressure can still penetrate the tissue, the expansion rate of the formed cavity may be lower than the injection flow rate of the jet. This leads to partial extrusion of the formulation. The favorable pressure combinations for a 1.0 mL injection volume, in terms of dispersion depth, are therefore (1.00–0.25) MPa or (1.25–0.25) MPa. These correspond to jet velocity combinations of (140.87–83.88) m/s and (153.88–83.88) m/s, respectively. Applying this analysis to the 1.5 mL condition yields favorable combinations of 1.25–0.25 MPa and 1.25–0.50 MPa, with corresponding velocities of 153.88–83.88 m/s and 153.88–104.74 m/s, respectively. For the 2.0 mL volume, the favorable combinations were (1.25–0.50) MPa and (1.50–0.50) MPa, yielding corresponding velocities of (153.88–104.74) m/s and (165.95–105.54) m/s, respectively. These results establish the favorable combinations of driving pressure and corresponding jet velocity for each injection volume, providing a basis for selecting parameters tailored to different clinical requirements.

### 2.4. Dispersion Width in Gel Injection

**Figure 8:**
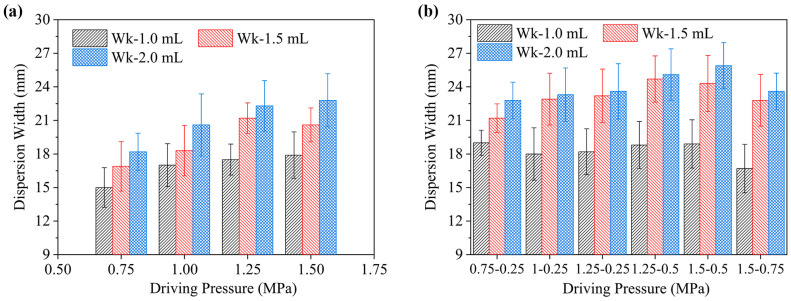
Dispersion width characteristics of two driving modes in a needle-free injection system under varying driving pressures and ejection volumes. (**a**) Single-phase driving mode; (**b**) two-phase driving mode.

Figure 8 presents the influence of driving pressure on the final dispersion width within the gel for both single-phase and two-phase driving modes, across ejection volumes of 1.0 to 2.0 mL. As shown in Figure 8a, for a 1.0 mL ejection volume under the single-phase mode, the dispersion width increases from 15.2 mm to 17.9 mm as the driving pressure rises from 0.75 MPa to 1.50 MPa, exhibiting an overall trend of initial increase followed by stabilization. A similar pattern is observed for the 1.5 mL volume, where the width stabilizes after the driving pressure reaches 1.25 MPa. For the 2.0 mL volume, however, the dispersion width maintains a continuous increasing trend with rising pressure.

In contrast, under the two-phase driving mode with a 1.0 mL volume (Figure 8b), dispersion widths of 19.12 mm, 18.13 mm, and 18.15 mm correspond to driving pressure combinations of (0.75–0.25) MPa, (1.00–0.25) MPa, and (1.25–0.25) MPa, respectively. These values are consistently greater than those achieved under the corresponding single-phase pressures (0.75, 1.00, and 1.25 MPa), indicating that the two-phase mode promotes more extensive lateral diffusion of the jet within the gel under equivalent initial (first-phase) driving pressures. Furthermore, analysis of the two-phase mode results (Figure 8b) reveals that the dispersion width exhibits a linear correlation with ejection volume under the (0.75–0.25) MPa pressure combination. However, under the (1.00–0.25) MPa combination, this correlation weakened and no significant difference was observed in the width between the 1.5 mL and 2.0 mL ejection volume. Notably, under these conditions, pronounced jet splash back onto the gel surface occurred during injection, indicating significant formulation loss. This emphasizes the importance of optimizing pressure combinations to achieve more controlled injection outcomes.

Further investigation was conducted under the 1.0 mL injection dose condition, revealing that with the second-phase pressure fixed at 0.25 MPa, an increase in the first-phase pressure from 0.75 MPa to 1.25 MPa resulted in an initial decrease in the dispersion width before stabilizing. Conversely, when the first-phase pressure was maintained at a constant level, while the second-phase pressure was increased from 0.25 MPa to 0.75 MPa, the resulting dispersion widths were measured to be 18.15 mm, 18.81 mm, 18.79 mm, and 16.70 mm, respectively. This data exhibited an initial increase, followed by a subsequent decrease. Consequently, to optimize dispersion width at a 1.0 mL ejection volume in two-phase mode, the suitable driving pressure combination is (1.25–0.25) MPa, corresponding to a jet velocity combination of (153.88–83.88) m/s. The employment of a consistent analytical approach enabled the determination of the favorable combination for a 1.5 mL ejection volume to be (1.25–0.50) MPa, with a corresponding velocity of (153.88–104.74) m/s. For a 2.0 mL ejection volume, the favorable combination was ascertained to be (1.50–0.50) MPa, with a corresponding velocity of (165.95–105.54) m/s.

### 2.5. Percent Delivery of Liquid Solution in Gel Injection

**Figure 9:**
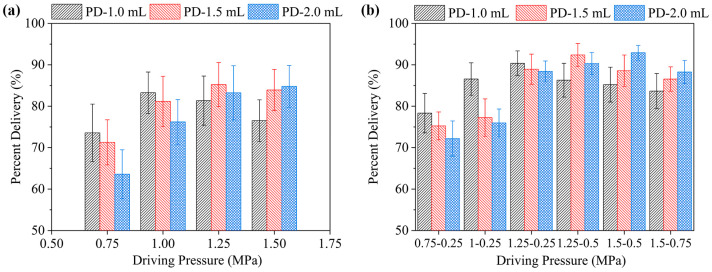
Percent delivery characteristics of two driving modes in a needle-free injection system under varying driving pressures and ejection volumes. (**a**) Single-phase driving mode; (**b**) two-phase driving mode.

As illustrated in Figure 9, the percentage of liquid drug delivered (PD) varies with driving pressure across different ejection volumes. In single-driving mode (Figure 9a), PD first increased and then decreased as driving pressure rose from 0.75 to 1.50 MPa for ejection volumes of 1.0 and 1.5 mL. The maximum values were 83.26% at a driving pressure of 1.00 MPa and 85.23% at a driving pressure of 1.25 MPa. At an injection volume of 2.0 mL, PD increased continuously with rising driving pressure, reaching a maximum of 84.79% at 1.50 MPa. These results suggest that increasing driving pressure improves drug delivery efficiency within the experimental parameters of single-drive mode, with a more significant improvement observed at larger ejection volumes. However, it is worth noting that the PD value remained below 90% across all single-driving modes. This may be because the jet forms cavities more slowly than the sustained ejection rate during large-volume (1.0–2.0 mL) injections. This results in a partial loss of the drug solution through cavitation backflow.

Figure 9b examines the effect of a two-phase driving pressure combination to further enhance delivery performance. The results show that using the two-phase driving mode increased the average PD to 84.86%, which is significantly higher than that using the single-phase driving mode. There was also reduced data variability. Concurrently, the maximum PD values corresponding to each ejection volume and their respective favorable driving pressure combinations can be identified. For an ejection volume of 1.0 mL, an PD of 90.37% is achieved at (1.25–0.25) MPa with corresponding jet velocities of (153.88–83.88) m/s. For the 1.5 mL and 2.0 mL ejection volumes, maximum PD values of 92.39% and 92.92% were achieved, respectively, under driving pressure combinations of (1.25–0.50) MPa and (1.50–0.50) MPa, corresponding to jet velocities of (153.88–104.74) m/s and (165.95–105.54) m/s respectively. These driving pressure combinations align with those identified as suitable in the results for dispersion depth and width. It is indicated that there are favorable driving pressure combinations (or jet velocity) for different ejection volumes that balance dispersion depth, dispersion width and the PD in the two-phase driving mode. This is particularly important when the ejection volume exceeds 1.0 mL, since increasing the jet velocity in both phases is necessary to deliver a high percentage of the liquid drug delivery in gels.

## 3. Conclusions

The present study systematically investigated the influence of single-phase and two-phase drive modes on the microjet flow characteristics of NFI. Based on these dynamic characteristics, it further examines the dynamic behavior and dispersion of microjets in tissue-mimicking agarose gels under large-volume (1.0–2.0 mL) conditions. The findings are as follows:

(1) The two-phase driving mode establishes a controllable “high-low” two-phase pressure profile, achieving the desired two-phase jet velocity via driving pressure switching. The jet velocity shows a linear positive correlation with the driving pressure in both phases, with the second-phase velocity being slightly higher than that of the single-phase mode under the same pressure due to residual pressure effects.
(2) The two-phase mode effectively optimizes the dispersion morphology of the jet within the gel. Compared to the single-phase mode, it not only eliminates jet splash-back but also enables stable drug delivery at a specific depth while promoting broader lateral diffusion, resulting in a dispersion profile closer to the ideal elliptical shape. The controllability and predictability of its dispersion behavior are validated by saturation exponential and segmented fitting models.
(3) The two-phase driving mode demonstrates significant advantages in regulating dispersion characteristics. Specifically, it enables the lateral diffusion of the jet stream once a specified diffusion depth has been reached. Furthermore, it maintains a consistent width advantage across all ejection volumes, producing a larger dispersion width than the single-phase mode. The relationship between driving pressure and width in this mode is nonlinear, following an initial increase and subsequent decrease, indicating the existence of a favorable driving pressure combination for maximizing lateral spread.
(4) The two-phase driving mode substantially increases the PD. Compared to single-phase mode, the average PD is significantly higher with reduced data variability. Analysis of liquid delivery percentages under different pressure combinations reveals maximum PD values of 90.37%, 92.39% and 92.92% for ejection volumes of 1.0, 1.5 and 2.0 mL, respectively. These values significantly outperform those obtained in single-phase mode (all below 90%).
(5) Favorable driving pressure and corresponding jet velocity combinations were identified for each ejection volume under the two-phase driving pressure mode: (1.25–0.25) MPa for 1.00 mL, (1.25–0.50) MPa for 1.50 mL, and (1.50–0.50) MPa for 2.00 mL, corresponding to jet velocity combinations of (153.88–83.88) m/s, (153.88–105.54) m/s, and (165.95–105.54) m/s, respectively. These results offer a theoretical foundation for selecting parameters tailored to specific clinical requirements.

In summary, the two-phase driving mode significantly improves the diffusion behavior of the jet within the simulated tissue gel model by enabling flexible adjustment of the pressure combination. The resulting shallower, wider elliptical diffusion pattern further reduces localized pressure and shear stress concentrations. This approach confines the high-pressure penetration phase to a brief initial period, with most of the volume delivery occurring under low pressure to promote extensive lateral diffusion. Consequently, the duration and intensity of tissue stress exposure are reduced, which are key biomechanical factors associated with tissue damage and nociceptor activation. This study establishes a crucial preclinical foundation for parameter optimization and paves the way for advancements in large-volume, needle-free injection systems. Future research should focus on achieving pressure-volume matching across broader parameter ranges in both in vitro and in vivo models, encompassing scenarios that reflect the diverse characteristics of biological tissues and drug properties, in order to comprehensively evaluate clinical applicability.

## 4. Materials and Methods

### 4.1. Experimental Setup

**Figure 10:**
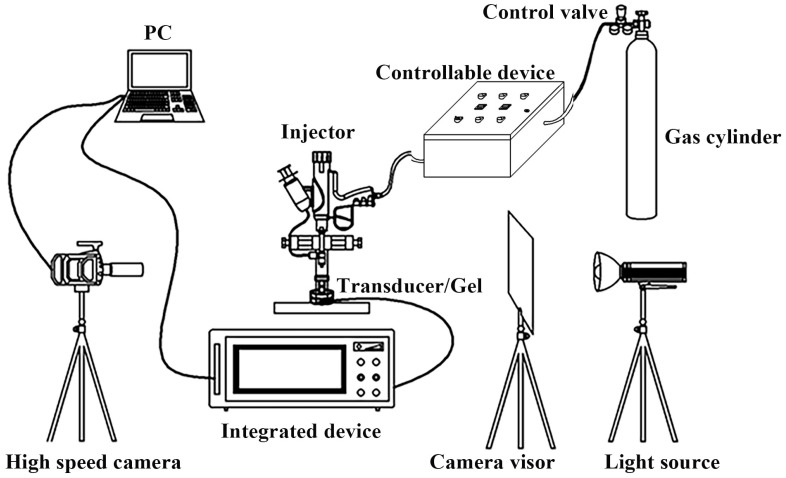
Schematic diagram of a high-speed camera device and pressure test platform for the needle-free injection.

A pneumatic needle-free injector described in prior work [16] was employed for injections of 1.0–2.0 mL through a 0.17 mm orifice under driving pressures ranging from 0.25 to 1.75 MPa. The integrated Laval nozzle amplifies the gas pressure by a factor of 9.55; thus, a driving pressure of 1.00 MPa yields a jet impact pressure of 9.55 MPa. To regulate jet velocity during larger-volume ejection, a rapid pressure-switching device was incorporated, enabling programmable transitions between high- and low-pressure phases. Both the pressure level and duration of each phase can be precisely set, and the device interfaces directly with existing pressure lines without hardware modification. Based on preliminary experiments and the time required for typical skin penetration documented, and ensuring a stable channel is established during the first phase, the duration of the first phase was set at 150 ms for a 1.0 mL ejection volume throughout this study. The overall experimental setup is illustrated in Figure 10.

An instrumented pressure-testing platform, detailed previously [22], was used to record the impact pressure at the nozzle outlet throughout injection. The platform comprises a Java-embedded control unit, a data acquisition system (HBM Quantum MX840B, HBM, Darmstadt, Germany), three dynamic pressure sensors (M5356 000002 040BG, MEAS, Guangzhou, China), and associated cabling. The target distance measured in this experiment was exceptionally short, meaning that the recorded pressure corresponds to the impact pressure within the core of the jet. Since the simplified Bernoulli equation is an estimate that incorporates assumptions, using it to convert pressure to velocity aligns with the measurement requirements for microjet impact pressure and velocity. The jet velocity at the nozzle exit was calculated from the measured impact pressure using the following equation [23]:

*P* = *ρ*_0_*v*^2^/2 = *F*/2*A*_0_ (7)

where *P* is the impact pressure at the nozzle outlet, *ρ*_0_ is the liquid density, *v* is the jet velocity, *F* is the impact force recorded by the sensor, and *A*_0_ is the cross-sectional area of the nozzle.

For gel injections, a high-speed video camera (Vision Research Phantom V2012, Vision Research, Inc., Wayne, NJ, USA) equipped with a Nikon ED AF Micro Nikkor 200 mm f/4 D lens (Nikon, Tokyo, Japan) captured the penetration process at 43,000 frames per second and an exposure time of 22 μs. Recorded videos were processed using Phantom Camera Control software (v2.0).

### 4.2. Materials

The injectate used in all experiments was distilled water. Its density (*ρ*_0_ ≈ 1000 kg/m^3^) and dynamic viscosity (≈1 mPa·s at 20 °C) are representative of many aqueous drug formulations. Transparent agarose gels were selected as tissue simulants due to their tunable mechanical properties, dimensional consistency, and optical clarity, which allow direct visualization of the injection process via high-speed photography [29]. Such gels are widely adopted for modeling skin in injection studies [30,31]. In this work, agarose gels (Biofroxx, Einhausen, Germany) with a mass fraction of 10% (Young’s modulus 12.875 ± 2.072 kPa) were prepared to mimic muscular tissue for investigating dispersion patterns under different driving modes [32,33]. Its viscosity ranges from 5 to 15 mPa·s at 40 °C in a 10% solution. Due to its viscoelastic and isotropic properties, the gel cannot fully replicate the mechanical characteristics of real skin tissue. Discrepancies in injection performance have been documented in previous studies [29,31]. Gel preparation involved mixing agarose powder with distilled water to the desired concentration, sealing the mixture, and heating it in a microwave oven in 30 s intervals until a clear solution formed. The solution was poured into a mold and allowed to solidify for 24 h before use.

### 4.3. Methods

**Figure 11:**
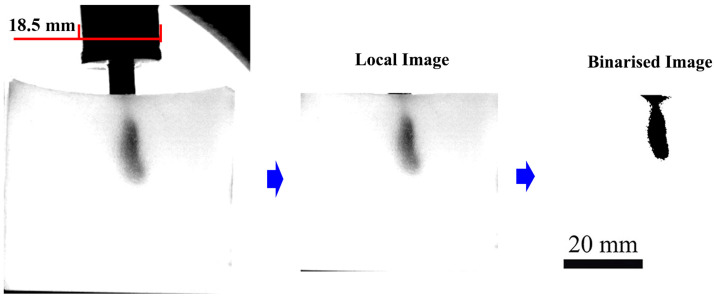
Schematic diagram of experimental results analysis methods for gel injections.

Spatial calibration was performed using the known diameter of the nozzle bottom contour (18.5 mm) as a reference; all captured images were scaled to a 20 mm calibration bar for quantitative analysis (Figure 11). To extract injection depth and spread width over time, the gel-injection images were first binarized in MATLAB (R2022a). Temporal profiles of depth and width were then obtained by calculating the corresponding pixel dimensions at each time point. Each set of experimental conditions was replicated five times. The percentage of liquid solution delivered was calculated by weighing the gel before and after injection. The data are presented as the mean ± standard deviation, based on a 95% confidence interval (*n* = 5).

## Data Availability

Data are contained within the article.

## Abbreviations

The following abbreviations are used in this manuscript:

NFI	Needle-free injection
*F*	The impact force
*P*	The nozzle outlet pressure
*r*	Jet radius
*v_jet_*	The jet velocity at the nozzle outlet
*ρ*_0_	The liquid density
*L_c_*	The total depth of dispersion
*W_k_*	The width of dispersion
*P*_1_	The impact pressure of the first phase
*P*_2_	The impact pressure of the second phase
*V*_1_	The jet velocity calculated by the impact pressure of the first phase
*V*_2_	The jet velocity calculated by the impact pressure of the second phase
*PD*	The percent delivery of liquid solution
